# Influence of In Vitro Gastric Digestion of Olive Leaf Extracts on Their Bioactive Properties against *H. pylori*

**DOI:** 10.3390/foods11131832

**Published:** 2022-06-22

**Authors:** Marisol Villalva, Jose Manuel Silvan, Esperanza Guerrero-Hurtado, Alba Gutierrez-Docio, Joaquín Navarro del Hierro, Teresa Alarcón-Cavero, Marin Prodanov, Diana Martin, Adolfo J. Martinez-Rodriguez

**Affiliations:** 1 Microbiology and Food Biocatalysis Group (MICROBIO), Department of Biotechnology and Food Microbiology, Institute of Food Science Research (CIAL, CSIC-UAM), C/Nicolás Cabrera, 9. Cantoblanco Campus, Autonomous University of Madrid, 28049 Madrid, Spain; marisol.villalva@csic.es (M.V.); jm.silvan@csic.es (J.M.S.); 2 Department of Production and Characterization of Novel Foods, Institute of Food Science Research (CIAL, CSIC-UAM), C/Nicolas Cabrera, 9. Cantoblanco Campus, Autonomous University of Madrid, 28049 Madrid, Spain; esperanza.guerrero@estudiante.uam.es (E.G.-H.); alba.gutierrez@uam.es (A.G.-D.); joaquin.navarrodel@uam.es (J.N.d.H.); marin.prodanov@uam.es (M.P.); diana.martin@uam.es (D.M.); 3 Microbiology Department, Hospital Universitario de La Princesa, Sanitaria Princesa Research Institute, 28006 Madrid, Spain; talarcon@helicobacterspain.com; 4 Department of Preventive Medicine, Public Health and Microbiology, School of Medicine, Autonomous University of Madrid, 28029 Madrid, Spain

**Keywords:** gastric digestion, olive leaf extract, *Helicobacter pylori*, anti-inflammatory activity, antioxidant activity, antibacterial activity, HPLC-PAD-MS characterization

## Abstract

The aim of this work was to evaluate the influence of in vitro gastric digestion of two olive leaf extracts (E1 and E2) on their chemical composition and bioactive properties against *Helicobacter pylori* (*H. pylori*), one of the most successful and prevalent human pathogens. HPLC-PAD/MS analysis and anti-inflammatory, antioxidant, and antibacterial activities of both olive leaf extracts were carried out before and after their in vitro gastric digestion. The results showed that gastric digestion produced modifications of the chemical composition and bioactive properties of both olive leaf extracts. The main compounds in the extract E1 were hydroxytyrosol and its glucoside derivatives (14,556 mg/100 g), presenting all the identified compounds a more polar character than those found in the E2 extract. E2 showed a higher concentration of less polar compounds than E1 extract, with oleuropein (21,419 mg/100 g) being the major component. Gastric digestion during the fasted state (pH 2) induced an overall decrease of the most identified compounds. In the extract E1, while the anti-inflammatory capacity showed only a slight decrease (9% of IL-8 production), the antioxidant properties suffered a drastic drop (23% of ROS inhibition), as well as the antibacterial capacity. However, in the extract E2, these changes caused an increase in the anti-inflammatory (19% of IL-8 production) and antioxidant activity (9% of ROS inhibition), which could be due to the hydrolysis of oleuropein and ligustroside into their main degradation products, hydroxytyrosol and tyrosol, but the antibacterial activity was reduced. Gastric digestion during fed state (pH 5) had less influence on the composition of the extracts, affecting in a lesser degree their anti-inflammatory and antioxidant activity, although there was a decrease in the antibacterial activity in both extracts similar to that observed at pH 2.

## 1. Introduction

*Helicobacter pylori* (*H. pylori*) is a Gram-negative bacterium found on the luminal surface of the gastric epithelium. It is one of the most successful and prevalent human pathogens that infect more than 50% of the world’s population [1,2]. *H. pylori* infection is recognized as a worldwide concern because it can cause several gastrointestinal disorders, including chronic active gastritis without clinical symptoms, as well as peptic ulceration, gastric adenocarcinoma, gastric mucosa-associated lymphoid tissue lymphoma, and other extra-gastric pathologies [2,3]. *H. pylori* has been classified as a type I carcinogen by the International Agency for Research on Cancer (IARC) [4] and it is considered the main infectious agent associated with cancer. In fact, the IARC estimated that 6.2% of all registered cancers were attributable to *H. pylori* [5].

Due to this high correlation between *H. pylori* infection and development of gastric cancer, most therapeutic international guidelines aim to eradicate this pathogen using a combination of antibiotics with a proton pump inhibitor in a triple or quadruple therapy [6,7]. However, the eradication rate of *H. pylori* treatment is markedly decreasing in recent years, mainly because the prevalence of antibiotic resistance strains appears to increase over time [8,9]. Failures in *H. pylori* eradication are alarming at present, but they are likely to be critical in the near future, since global rates of antibiotic resistance are rising, and *H. pylori* therapy is increasingly prescribed. This fact and the relationship that has been observed between the eradication of *H. pylori* and the onset or worsening of other pathologies, such as esophageal reflux, has led many researchers to question whether it is always essential to carry out an eradicative therapy, even in the absence of symptoms, or whether it is possible to consider milder and more sustainable treatments to fight against *H. pylori* although they do not have the eradicating power of antibiotics [10]. Therefore, the use of alternative therapies that could enhance antibiotic activity or modulate inflammatory response to support *H. pylori* eradicative therapy appears to be reasonable. In this regard, extracts with bioactive properties obtained from food ingredients or food by-products are showed as potentially useful candidates in the complementary therapy against *H. pylori* [11,12,13,14,15].

Olive industry by-products (including olive mill wastewaters, olive pomace, and olive leaves) are considered a relevant source of bioactive compounds and olive leaves represent 10% of the total weight of the olives arriving at mills, containing considerable amounts of bioactive secondary metabolites which have particular importance in their health properties [16]. Recently, we have reported that olive leaf extracts with different composition showed anti-inflammatory, antioxidant, and antibacterial activity against *H. pylori*. These properties were significantly related with the phenolic and secoiridoid composition of these extracts [13], which can vary depending on factors, such as olive variety and growing, climatic, and storage conditions. Oleuropein and hydroxytyrosol are mayor constituents of olive leaf extracts, followed by verbascoside, apigenin-7-glucoside, luteolin-7-glucoside, and tyrosol. These compounds have been related to the anti-inflammatory, antioxidant, and antibacterial properties associated to olive leaf extracts [17,18,19]. Because *H. pylori* has developed acid acclimation mechanisms to colonize the acidic gastric niche, effective bioactive compounds against this bacterium should keep their stability at gastric conditions. It has been described that the individual stability of the main phenolic compounds present in olive leaf extracts may be affected during gastrointestinal digestion by the combined effect of enzymatic activity and pH changes [20,21,22]. Since the modification of bioactive compounds during gastric digestion has been mainly related to the acid conditions of the media, it is relevant to consider that the specific values of acid pH of the gastric environment are quite dynamic depending on the type and amount of the intake. In general, gastric pH increases from around 1–2 (fasted state) to around 5–7 (fed state), just after food ingestion, and it depends on the buffering capacity or the solid/liquid state of the meal [23]. Therefore, the evaluation of the impact of gastric digestion on the bioactivity of olive leaf extracts would be relevant in order to establish the efficacy of these extracts at the specific point of action, but also to further evidence the optimal intake patterns to take full advantage of its effectiveness. In this sense, different static in vitro models simulating gastrointestinal conditions have been developed as suitable, inexpensive, and simple alternatives to in vivo methods to assess the effect of the digestion process on the stability of bioactive compounds. In static in vitro digestion models, samples are subjected to a sequential digestion in which the oral, gastric, and intestinal phases are simulated. Briefly, these are performed by including the experimental product in a buffer solution containing different salts and the digestive components of each phase, mainly enzymes (amylase for oral, pepsin and lipase for gastric, and a pool of amylase, protease, and lipase for intestinal digestions), as well as a bile solution in case of intestinal digestion. The buffer solution allows to keep the specific pH for each phase and to simulate the main aqueous environment of the gastrointestinal tract. Then, conditions of shaking or mixing and physiological temperatures are reproduced for a variable time, depending on the phase (up to 2 min for oral, 1–3 h for gastric, or 1–5 h for intestinal digestion) [24,25,26]. Additionally, the versatility of these models allows to easily test the digestion process under different physiological conditions, as fed and fasted states might be.

Therefore, the aim of the present work was to evaluate the impact of the gastric digestion of two olive leaf extracts with different phenolic compositions on the anti-inflammatory, antioxidant, and antibacterial activity against *H. pylori*. With this purpose, in vitro gastric digestions were simulated under typical pH values of fed (pH 5) and fasted (pH 2) states and the subsequent changes of the phenolic and secoiridoid profiles and bioactivities were established.

## 2. Materials and Methods

### 2.1. Olive Leaf Extracts and Chemicals

Extract E1 was standardized in 4% elenolic acid and its derivates (Isenolic^®^) and extract E2 was standardized in 20% of oleuropein (Olivactive^®^). They were provided by Pharmactive Biotech Products S.L. (Madrid, Spain). The following reference substances *trans*-4,5-DCQA (*trans*-4,5-dicaffeoylquinic acid) (>95%), quercetin (>95%), 4-HPE-EA-glucoside (ligustroside) (>96.2%), and 3,4-DHPE-EA-glucoside (oleuropein) (>98%) were acquired from Merck (Dramstadt, Germany). Elenolic acid (EA) (>98%) and luteolin (>95%) were purchased from Toronto Research Chemicals (Toronto, ON, Canada). The reference substances 3,4-DHBA (protocatechuic acid) (>90%), 4-HPE (tyrosol) (>95%), *trans*-3,4-DHCA (*trans*-caffeic acid) (>99%), *trans*-4-HCA (*trans*-4-coumaric acid) (>98%), *trans*-3-M,4-HCA (*trans*-ferulic acid) (>98%), quercetin 3-*O*-rhamnoside (quercitrin) (>93.3%), luteolin 3′,7-di-*O*-glucoside (>97%), eriodictyol-7-*O*-rutinoside (>98%), eriodictyol 7-*O*-glucoside (>98%), luteolin 7-*O*-glucoside (>98%), and 3,4-DHPE caffeoyl glucoside (verbascoside) (>95%) were obtained from Extrasynthese (Genay, France). EA 2-glucoside (oleoside 11-methyl ester) (>98%), EA monoaldehyde form (EMA) 2-glucoside (secoxyloganin) (>99%), 3,4-DHPE (Hydroxytyrosol) (>90%), quercetin 3-*O*-glucoside (isoquercitrin) (>99%), apigenin 7-*O*-glucuronide (>90%), and luteolin 4′-methyl ether 7-*O*-glucoside (diosmin) (>90%) were purchased from PhytoLab GmbH & Co. KG (Vestenbergsgreuth, Germany). Apigenin 6,8-di-C-glucoside (>95%) was obtained from Glentham Life Sciences (Corsham, UK). Apigenin 7-*O*-rutinoside (isorhoifolin) (>99.9%) was obtained from Biosynth AG (Staad, Switzerland). The substance 3,4-dihydroxyphenylglycol (3,4-DHPG) (75%) was provided by Prof. Juan Fernández-Bolaños from Instituto de la Grasa (CSIC) (Sevilla, Spain). Monohydrate extra pure (99.5%) reference 3,4,5-THBA (gallic acid) was purchased from Scharlab (Barcelona, Spain).

### 2.2. In Vitro Simulated Gastric Digestion of Olive Leaf Extracts

The in vitro simulated gastric digestion was based on the gastrointestinal digestion model described by Navarro del Hierro et al. with minor modifications [27]. Briefly, extracts (E1 and E2) were solubilized in 10 mg/mL of gastric solution (150 mM NaCl, 6 mM CaCl_2_) adjusted at pH 2 (fasted state) (DE1 and DE2 pH2) or pH 5 (fed state) (DE1 and DE2 pH5) with HCl (4 mM). This gastric solution contained pepsin at 0.85 mg/mL, which was previously prepared by stirring for 10 min. Digestions were performed in an orbital incubator (Titramax 1000, Heidolph Instruments, Schwabach, Germany) at 37 °C and the gastric digestion was performed for 120 min at 250 rpm. Once finished, samples were stored at −20 °C until use. Digestions for each extract at each pH were performed in duplicate (n = 2). Control digestion media at each tested pH and in absence of extracts were also prepared for the bioactivity assays.

**Figure 1:**
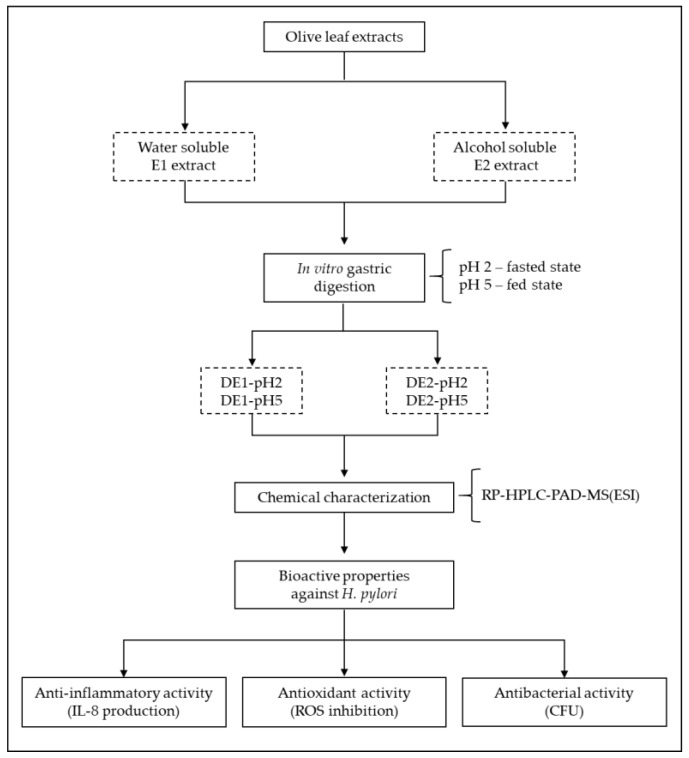
Flowchart summarizing the main experimental procedures for evaluation of the bioactive properties of olive leaf extracts and their gastric digests.

A graphical flowchart summarizing the main steps of the experimental procedure applied is provided in Figure 1.

### 2.3. Chemical Characterization of Olive Leaf Extracts and Their Gastric Digests

Solutions of 10 mg/mL of water soluble E1 and alcohol soluble E2 extracts and their gastric digests (DE1 and DE2) at different pH were prepared in duplicate (n = 2), in water and methanol, respectively, and were analyzed by reverse-phase high performance liquid chromatography (RP-HPLC), coupled sequentially to photodiode array detector (PAD) and mass spectrometry (MS) with electrospray ionization source (RP-HPLC-PAD-MS(ESI)), as described by Silvan et al. [13].

The 3,4-DHBA, 3,4-DHPE, 4-HPE, 3,4-DHPE-EA-glucoside, 3,4-DHPE caffeoyl glucoside, quercetin, quercetin 3-*O*-glucoside, apigenin 7-*O*-glucuronide, apigenin 6,8-di-C-glucoside, luteolin, luteolin 3′,7-di-*O*-glucoside, luteolin 7-*O*-glucoside, luteolin 4′-*O*-methyl ether 7-*O*-glucoside, eriodictyol 7-*O*-rutinoside, EA, EA 2-glucoside, EMA 2-glucoside, *trans*-3,4-DHCA, *trans*-4-HCA, *trans*-3-M,4-HCA, *trans*-4,5-DCQA, and 3,4,5-THBA were identified unambiguously by co-elution and comparison with their retention time, order of elution, UV spectra, and pseudo-molecular and fragment ion masses of the corresponding reference substances, and quantified according to the calibration curves of each of them. The glucosides of 3,4-DHBA, 3,4-DHPE, and 3,4,5-THBA were identified tentatively by using their corresponding retention time, order of elution, UV spectra, pseudo-molecular, diagnostic fragment ion masses, and bibliographic data [13]. The 3,4-DHBA glucoside was quantified as equivalents of 3,4-DHBA, the three 3,4-DHPE glucosides were quantified as equivalents of 3,4-DHPE, and the two 3,4,5-THBA glucosides were quantified as equivalents of 3,4,5-THBA.

### 2.4. Helicobacter pylori, Growth Media and Culture Conditions

*H. pylori* strain (HpCIAL2) was obtained from MICROBIO bacterial collection (CIAL-CSIC) by isolation from a gastric biopsy in the Princesa Universitary Hospital, Madrid, Spain. *H. pylori* strain was stored at −80 °C in Brucella Broth (BB) (Becton, Dickinson, & Co., Madrid, Spain) plus 20% glycerol. The agar-plating medium used was Müeller-Hinton agar supplemented with 5% defibrinated sheep blood (MHB) (Becton, Dickinson, & Co, Madrid, Spain). Liquid medium BB supplemented with 10% horse serum (HS) (Biowest, Barcelona, Spain) was used. The inoculum for *H. pylori* strain was prepared as described by Silvan et al. (2020) [11] and it can be summarized as follows: frozen strain was reactivated by inoculation (200 μL) in MHB plate and incubation in a Variable Atmosphere Incubator (VAIN) (85% N_2_, 10% CO_2_ and 5% O_2_) (MACS-VA500, Don Whitley Scientific, Bingley, UK) at 37 °C for 72 h. Bacterial grown from one MHB plate was suspended in 2 mL of BB + 10% HS or culture medium cell (~1 × 10^8^ colony forming units (CFU/mL)) and used as bacterial inoculum in the different assays.

### 2.5. Human Gastric Epithelial Cell Cultures

AGS cells (human gastric epithelial cell line) were obtained from the American Type Culture Collection (ATCC). Cells were cultured in Dulbecco’s Modified Eagle’s Medium F12 (DMEM/F12) (Lonza, Madrid, Spain) supplemented with 10% fetal bovine serum (FBS) (Hyclone, GE Healthcare, Logan, UK) and 1% penicillin/streptomycin (5000 U/mL) (Lonza). Cell cultures and subcultures were prepared as described by Silvan et al. [12]. Briefly, cells were plated at densities of ~1 × 10^6^ cells in 75 cm^2^ culture flasks (Sarstedt, Barcelona, Spain) and maintained at 37 °C under 5% CO_2_ in a humidified incubator until 90% of cell confluence. The cell culture medium was changed every 2 days. Before a confluent monolayer appeared, cell sub-culturing was carried out. All experiments were carried out between passage 10 and passage 30 to ensure cell uniformity and reproducibility.

### 2.6. Cell Viability

Previously to the antioxidant and anti-inflammatory assays, cytotoxicity of olive leaf extracts and gastric digested samples was evaluated. AGS cell viability was determined by MTT (3,4,5-dimethylthiazol-2,5-diphenyl-tetrazolium bromide) (Sigma, Madrid, Spain) reduction assay following a protocol previously described [13]. Briefly, confluent cell cultures (~90%) were trypsinized (Trypsin/EDTA 170,000 U/L) (Lonza) and cells were seeded (~5 × 10^4^ cells per well) in 96-well plates (Sarstedt) and incubated in culture medium at 37 °C under 5% CO_2_ in a humidifier incubator for 24 h. Cell culture medium was replaced with serum-free medium containing the non-digested and digested extracts (at 2 mg/mL final concentration) and cells were incubated at 37 °C under 5% CO_2_ for 2 h. Control cells (non-treated) were incubated in serum-free medium without extracts. Thereafter, cells were washed with phosphate buffered saline (PBS) (Lonza), the medium was replaced by 200 μL of serum-free medium, and 20 μL of MTT solution in PBS (5 mg/mL) was added to each well for the quantification of the living metabolically active cells after 1-h incubation. During this period, MTT is reduced to purple formazan in the mitochondria of living cells. Formazan crystals in the wells were solubilized in 200 μL dimethyl sulfoxide (Sigma, Madrid, Spain). Finally, absorbance was measured at 570 nm wavelength using a microplate reader Synergy HT (BioTek Instruments Inc., Winooski, VT, USA). The viability was calculated considering controls containing serum-free medium (non-treated cells) as 100% viable. Results were obtained from three independent experiments (n = 3).

### 2.7. Determination of Anti-Inflammatory Activity of Olive Leaf Extracts and Their Gastric Digests after Infection of AGS Human Gastric Epithelial Cells with H. pylori

The assay was carried out following the procedure described by Silvan et al. [28]. Briefly, human gastric AGS cells were seeded (~5 × 10^4^ cells/well) in 24-well plates (Sarstedt) and incubated in cell culture medium at 37 °C under 5% CO^2^ in a humidifier incubator until a monolayer was formed. Cells were incubated with non-digested (E1 and E2) and digested olive leaf extracts (DE1 and DE2 at pH 2 and pH 5) at 37 °C under 5% CO_2_ for 2 h. The inflammatory response was determined using 2 mg/mL of each extract, since this concentration results as non-cytotoxic for AGS cells during viability test (data not shown). Cells were washed twice with PBS and infected with 0.5 mL/well of *H. pylori* inoculum in serum-antibiotics-free medium (~1 × 10^8^ CFU/mL for all tested strains). The cells and bacteria were incubated at 37 °C under 5% CO_2_ for 24 h to allow the bacteria to adhere to the gastric cells. Uninfected cells were included in the experiment as a control. At the end of incubation, the cell supernatants were collected, particulate material was removed by centrifugation (12,000 rpm, 10 min), and samples were stored at −20 °C until analysis were performed. The amounts of secreted IL-8 cytokine in the collected supernatant of gastric AGS cell samples were determined by ELISA assay. Commercially available ELISA kit (Diaclone) for the quantitation of IL-8 cytokine was used as described in the manufacturer’s instructions. The absorbance was measured at 450 nm using a microplate reader Synergy HT (BioTek Instruments Inc. Winooski, VT, USA). Such as in the absence of bacteria, gastric cells release small amounts of IL-8 [28], so titers of cytokine released by AGS cells were determined experimentally. Results were expressed as IL-8 production (pg/mL) of three independent experiments (n = 3).

### 2.8. Determination of Antioxidant Activity of Olive Leaf Extracts and Their Gastric Digests against Intracellular Reactive Oxygen Species (ROS) Production in AGS Cells

Human gastric AGS cells were used for the evaluation of oxidative stress induced by *H. pylori* infection. Intracellular ROS were measured by the DCFH-DA (carboxy-2′,7′-dichloro-dihydrofluorescein diacetate) (Sigma) assay as was previously reported by Silvan et al. [13]. Cells were seeded (5 × 10^4^ cells per well) in 24-well plates and grown until they reached 70% of confluence. Cells were incubated with non-digested (E1 and E2) and digested olive leaf extracts (DE1 and DE2 at pH 2 and pH 5) (2 mg/mL) dissolved in serum-free medium at 37 °C under 5% CO_2_ for 2 h. After that, cells were washed with PBS and incubated with 20 μM DCFH-DA at 37 °C under 5% CO_2_ for 30 min. Then, cells were washed twice with PBS to remove the unabsorbed probe and infected with *H. pylori* inoculum strain suspended in serum-antibiotics-free medium (~1 × 10^8^ CFU/mL). ROS production was immediately monitored for 180 min in a fluorescent microplate reader Synergy HT (BioTek Instruments Inc. Winooski, VT, USA) using a λ_ex_ 485 nm and λ_em_ 530 nm. After being oxidized by intracellular oxidants, DCFH-DA changes to dichlorofluorescein (DCF) and emits fluorescence. Non-treated but infected cells were used as oxidative control (100% of intracellular ROS production). All samples were analyzed in triplicate (n = 3). Results were expressed as % of inhibition of ROS production.

### 2.9. Determination of Antibacterial Activity of Olive Leaf Extracts and Their Gastric Digests against H. pylori

The antibacterial activity of non-digested (E1 and E2) and digested olive leaf extracts (DE1 and DE2 at pH 2 and pH 5) against *H. pylori* strain was evaluated following the procedure described by Silvan et al. [29]. Briefly, 1 mL of extracts (at 2 mg/mL final concentration) was transferred into different flasks containing 4 mL of BB supplemented with 10% HS. Bacterial inoculum (100 μL of ~1 × 10^8^ CFU/mL) was then inoculated into the flasks under aseptic conditions. The cultures were incubated under stirring (150 rpm) in a microaerophilic atmosphere using a VAIN at 37 °C for 24 h. Growth controls were prepared by transferring 1 mL of sterile water to 4 mL of BB supplemented with 10% HS and 50 μL of bacterial inoculum. After incubation, serial decimal dilutions of cultures were prepared in saline solution (0.9% NaCl) and they were plated (20 μL) onto fresh MHB agar, and incubated in a microaerophilic atmosphere using a VAIN at 37 °C for 72 h. The number of CFU was assessed after incubation. The results of antibacterial activity were expressed as log CFU/mL (n = 3).

### 2.10. Statistical Analysis

The results were reported as mean value ± standard deviations (SD) performed at least in triplicate (n = 3). Statistical analyses of the concentration of each quantified compound in both olive leaf extracts were performed by *t*-test. Significant differences in the anti-inflammatory and antioxidant activity results were estimated by applying analysis of variance (ANOVA). The Tukey’s least significant differences (HSD) test was used to evaluate the significance of these values. In all cases, differences were considered significant at *p* < 0.05. All statistical tests were performed with IBM SPSS software statistics for Windows, Version 26.0 (IBM Corp., Armonk, NY, USA).

## 3. Results and Discussion

### 3.1. Effect of In Vitro Gastric Digestion on the Chemical Composition of Olive Leaf Extracts

Results of the chemical composition and quantitative HPLC-PAD-MS determination of phenolic and secoiridoid compounds and their derivatives in the non-digested and gastric digested extracts are shown in Table 1 (date is in Supplementary Figure S1 and S2).

A whole of 24 phenolic, secoiridoid, and their derivative compounds were identified in extract E1 and its gastric digested extracts (DE1) at pH 2 and pH 5. They could be divided into seven groups of compounds: hydroxybenzoic acids, hydroxycinnamic acids, phenylethanols, secoiridoids, secoiridoid phenylethanols, cinnamoyl phenylethanols, and flavones. One of the most remarkable characteristics of olive leaf extract E1 was its high content in phenylethanols and their glycoside derivatives (15,237 mg/100 g), such as hydroxytyrosol and three of its glucosides (14,556 mg/100 g), as well as relevant amounts of elenolic acid and its glucoside derivatives (2526 mg/100 g), and flavones (1341 mg/100 g), such as luteolin 7-*O*-glucoside (702 mg/100 g). Gastric digestion of extract E1 at pH 2 (DE1-pH2) showed an overall decrease of most of the identified compounds, which was low to moderate for the protocatechuic acid glucoside (14%), all hydroxycinnamic acids and derivatives (16–25%), all phenylethanols and glycoside derivatives (14–21%), all secoiridoids and glycoside derivatives (18–27%), verbascoside (16%), and for some flavones, such as both apigenin (20%) and luteolin (12%), diglucosides, and luteolin 4′-methyl ether 7-*O*-glucoside (19%). A greater decrease of the contents of protocatechuic acid (36%), oleuropein (34%), and ligustroside (41%), and some flavones, such as apigenin 7-*O*-rutinoside (34%), apigenin 7-*O*-glucuronide (51%), and luteolin 7-*O*-glucoside (45%), was obtained. The largest drop was found for the contents of both galloyl glucosides (1 and 2), which were completely hydrolyzed at pH 2 to free gallic acid. In this case, the amount of gallic acid raised up to 86.1 mg/100 g. The total concentration of phenolic and secoiridoid compounds in extract E1 dropped by 18% during gastric digestion at pH 2. This value was rather lower than that reported by others (35–70%) for extracts derived from olive oil by-products [30,31]. This may be due to the fact that hydroxytyrosol was the major compound in extract E1 (66% of total phenolic and secoiridoid compounds), which together with its glucoside derivatives are quite stable to acidic gastric digestion conditions [32]. Gastric digestion of extract E1 at pH 5 (DE1-pH5) did not produce significant differences (*p* > 0.05) for the contents of most of the identified compounds. Nevertheless, a slight decrease of verbascoside content (15%) and a greater decrease of elenolic acid content (65%) was observed. Similar to DE1-pH2, gallic acid appeared in DE1-pH5 (37.2 mg/100 g). The partial hydrolysis of the labile linkage between the gallic acid and glucose moiety of both galloyl glucosides at the moderate acidic conditions of DE1-pH5 [33] could partly explain the emergence of gallic acid. Extraction of negative *m/z* 331 ion of the non-digested extract E1 that corresponds to the mass of monogalloyl glucosides gave six peaks with different intensities (data not shown) and extraction of ions at *m/z* 483 and 493, corresponding to digalloyl glucosides and monogalloyl diglucosides, respectively, also gave two peaks. In this work, only two monogalloyl glucosides were identified and quantified, but the other unidentified galloyl compounds should also contribute to the total amount of gallic acid in extract DE1-pH5.

On the other hand, 25 phenolic, secoiridoid, and their derivative compounds were identified in olive leaf extract E2 and its gastric digested extracts (DE2) at pH 2 and pH 5, which can be divided into nine groups: hydroxybenzoic acids, hydroxycinnamic acids, phenylethanols, secoiridoids, secoiridoid phenylethanols, cinnamoyl phenylethanols, flavones, flavonols, and flavanones. Extract E2 was characterized mainly by its high content in oleuropein (21,419 mg/100 g) and verbascoside (7255 mg/100 g). Flavones were also detected in relevant amounts (924 mg/100 g), mainly luteolin 7-*O*-glucoside (517 mg/100 g) and apigenin 7-*O*-rutinoside (124 mg/100 g). It also contained several flavonol and flavanone compounds that were not detected in extract E1. Galloyl glucosides and some secoiridoids, such as elenolic acid and secoxyloganin, were not detected in extract E2. Gastric digestion of extract E2 at pH 2 (DE2-pH2) increased significantly (*p* < 0.05) the content of all phenylethanols and their glucoside derivatives. Among them, hydroxytyrosol and tyrosol sharply rose with 1342% and 277%, respectively. This behavior is related to the hydrolysis of oleuropein and ligustroside [34], whose contents decreased by 66% and 54%, respectively. This result may confirm the possible hydrolysis of oleuropein and ligustroside and probably other isomers and derivatives that were not identified in the present work. Oleuropein, which is usually the major component of many other olive leaf extracts, was reported as highly sensitive to digestive degradation [35,36]. However, a proportional increase in the amounts of elenolic acid and/or its glucosides was not observed, suggesting that this increment could be related with the partial hydrolysis of verbascoside or other hydroxytyrosol/tyrosol containing unidentified compounds, such as oleuropein/ligustroside isomers and hydroxytyrosol/tyrosol elenolate [37], also known as oleuropein/ligustroside aglycons. A small increase of quercetin (6%) was also observed, due most probably to a possible acid hydrolysis of the flavonols quercitrin and isoquercitrin [38]. Similar to gastric digestion of E1 extract, most of the identified compounds decreased significantly (*p* < 0.05) during digestion at pH 2. Protocatechuic acid (23%), most of the hydroxycinnamic acids (9–24%), and luteolin 3′,7-di-*O*-glucoside (10%) decreased slightly to moderately. The most affected compounds were *trans*-4-coumaric acid (40%), oleoside 11-methyl ester (72%), verbascoside (28%), most flavones (38–74%), both flavonol glycosides, quercitrin (55%) and isoquercitrin (56%), as well as both flavanones, eriodictyol 7-*O*-rutinoside and eriodictyol 7-*O*-glucoside (44% and 100%, respectively). Gastric digestion of extract E2 at pH 5 (DE2-pH5) had a similar behavior to extract E1. It was observed no significant (*p* > 0.05) changes or a little decrease in the contents of protocatechuic, most of the hydroxycinnamic acids, phenylethanols, oleuropein, ligustroside, verbascoside, most flavones, all flavonols, and both flavanones, eriodictyol 7-*O*-rutinoside and eriodictyol 7-*O*-glucoside. However, the content of some compounds, such as *trans*-4-coumaric acid (38%), oleoside 11-methyl ester (42%), apigenin 7-*O*-glucuronide (59%), and luteolin 4′-methyl ether 7-*O*-glucoside (34%), were significantly (*p* < 0.05) decreased.

### 3.2. Effect of In Vitro Gastric Digestion of Olive Leaf Extracts on Their Anti-Inflammatory Properties in H. pylori-Infected AGS Cells

**Figure 2:**
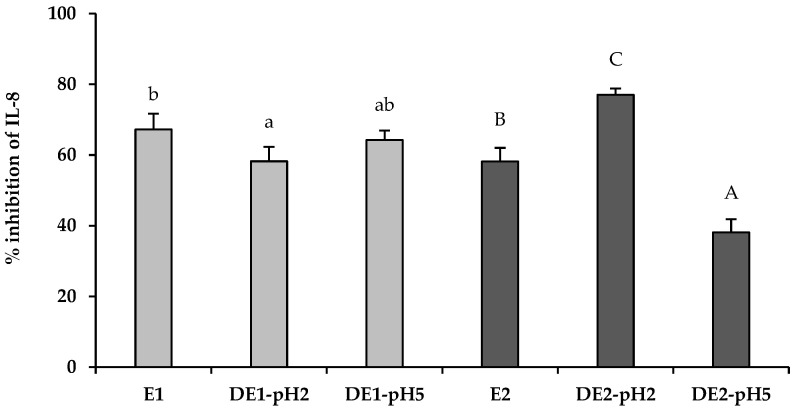
Inhibition effect of olive leaf extracts (E1 and E2) and their gastric digests at pH 2 and pH 5 (2 mg/mL) on pro-inflammatory cytokine IL-8 production by human gastric epithelial AGS cells after *H. pylori* infection. The experimental control (AGS cell without extracts) had 0% of IL-8 inhibition (data not showed). Values are the mean ± SD (n = 3). ^a,b, ab, A,B,C^ Different letters indicate statistical difference within a same extract by ANOVA post hoc HSD Tukey test (*p* < 0.05).

As shown in Figure 2, when AGS cells were pretreated for 2 h with the non-digested olive leaf extracts (E1 and E2) and subsequently gastric cells were infected with *H. pylori*, both extracts exerted a relevant inhibition of IL-8 pro-inflammatory cytokine production (67% and 58% of inhibition, respectively) with respect to the untreated infected control group. It is noteworthy that extract E1 exhibited slightly improved anti-inflammatory activity than was shown by extract E2. In addition, although both extracts were submitted to a gastric digestion with different pH conditions (pH 2 and pH 5), the anti-inflammatory effect was significant (*p* < 0.05) for all of the digested samples with respect to the untreated infected control group. Individually, when extract E1 was digested at pH 2, the anti-inflammatory effect was significantly (*p* < 0.05) reduced with respect to that shown by the non-digested extract E1, but nevertheless the IL-8 secretion was inhibited up to 58%; however, when extract E1 was digested at pH 5, the inhibition of IL-8 (64%) was remained similar to that exhibited by the non-digested extract E1. The decrease of this bioactivity in DE1-pH2 was consistent with a reduction in the concentration of most of the determined phenolic and secoiridoid compounds (Table 1). Among them, potent anti-inflammatory activity has been described for the major compounds present in E1 that decrease significantly after gastric digestion, such as hydroxytyrosol [39], elenolic acid [40], and luteolin-7-glycoside [41]. Regarding the extract E2, when it was digested at pH 2, the inhibition effect on the IL-8 production was significantly (*p* < 0.05) higher (77%) compared to that observed with the non-digested extract E2 (58%). This effect coincides with oleuropein degradation and the increase in hydroxytyrosol and tyrosol concentration (Table 1). Others previously reported the significant anti-inflammatory properties of these compounds [39,41]. Conversely, the inhibition effect on IL-8 production was significantly reduced (*p* < 0.05) when extract E2 was digested at pH 5 (38%) with respect to extract E2 before digestion (58%). In this case, the milder digestion conditions do not cause extensive degradation of oleuropein, and therefore, the concentration of hydroxytyrosol and tyrosol does not increase as in the pH2 digestion.

### 3.3. Effect of the In Vitro Gastric Digestion of Olive Leaf Extracts on Their Antioxidant Activity against Intracellular ROS Production in H. pylori-Infected AGS Cells

**Figure 3:**
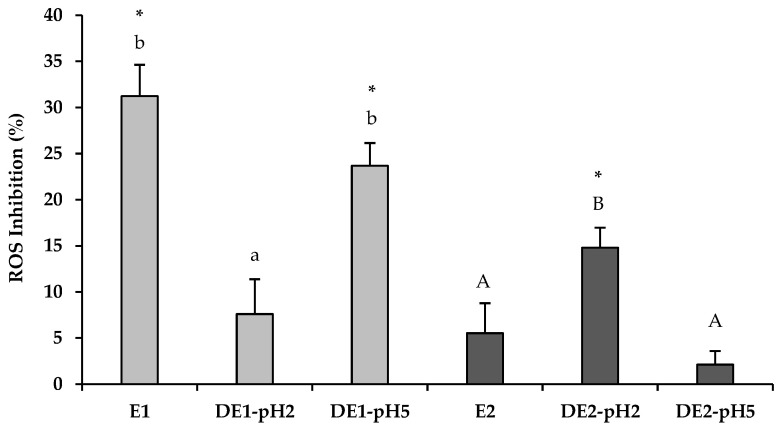
Inhibition effect of olive leaf extracts (E1 and E2) and their gastric digests at pH 2 and pH 5 (2 mg/mL) on intracellular reactive oxygen species (ROS) production by human gastric epithelial AGS cells after *H. pylori* infection. The experimental control (AGS cell without extracts) had 0% of ROS inhibition (data not showed). Values are the mean ± SD (n = 3). Asterisk indicate significant differences compared to the control group (untreated infected AGS cells) by *t*-test (*p* < 0.05). ^a,b,A,B^ Different letters indicate statistical difference within a same extract by ANOVA post hoc HSD Tukey test (*p* < 0.05).

Figure 3 shows the inhibition of intracellular ROS production in *H. pylori*-infected AGS cells obtained after pre-treatment with olive leaf extracts, non-digested and gastric digested at pH 2 and pH 5, respectively. Results showed a similar behavior to those obtained in the analysis of anti-inflammatory activity. Non-digested extract E1 presented the most potent antioxidant activity (31% of inhibition), but significantly (*p* < 0.05) decreased to 8% of ROS inhibition after gastric digestion at pH 2. In contrast, when the pH of gastric digestion was set at 5, the antioxidant activity of digested extract (DE1-pH5) was close to 24% of ROS inhibition. On the other hand, extract E2 before gastric digestion showed no significant effect (*p* > 0.05) on ROS inhibition (5%) with respect to the control group (untreated infected AGS cells). However, after gastric digestion at pH 2 (DE2-pH2), ROS inhibition capacity was significantly (*p* < 0.05) increased up to 15%. In contrast to the extract E1, digestion of extract E2 at pH 2 resulted in an increase of anti-inflammatory and antioxidant activity with respect to non-digested extract E2. This behavior was probably related with the synergistic effect between oleuropein and its degradation products (hydroxytyrosol and tyrosol), since all these compounds have been previously related to the bioactivities described in this work [13,35,39,42]. It has been reported that the effects of oleuropein and hydroxytyrosol on ROS generation by stimulated neutrophils are mainly due to scavenging of ROS. More specifically, they can scavenge hydrogen peroxide [43,44]. Finally, when extract E2 was submitted to gastric digestion at pH 5, ROS inhibition (2%) remained at a similar capacity to the non-digested extract E2. As mentioned above, under these digestion conditions, there is no extensive degradation of oleuropein, and therefore, the concentration of hydroxytyrosol and tyrosol does not increase as in the pH2 digestion.

### 3.4. Effect of the In Vitro Gastric Digestion of Olive Leaf Extracts on Their Antibacterial Activity against H. pylori

The antibacterial activity of olive leaf extracts, E1 and E2, as well as their gastric digested samples is presented in Table 2. As can be observed, different levels of bacterial growth inhibition were shown. Non-digested extract E1 significantly (*p* < 0.05) inhibited the growth of *H. pylori* strain. Reduction of log CFU was 1.6, being the higher reduction achieved in this assay. After E1 gastric digestion, regardless of the pH used, a decrease in antibacterial activity was observed of the same extent (log CFU of 0.5). As the number of compounds that decreased during gastric digestion of E1 at fed state was limited, these results suggest that other important components, such as elenolic acid (which decreased by 65%) and verbascoside (15%), may be involved in this activity. The antibacterial activity of different extracts from olive has been associated with the presence of these compounds [45,46]. On the other hand, the activity of the non-digested extract E2 was lower than that obtained for extract E1, since just a reduction of 0.6 CFU was observed. The reduction on antibacterial properties of extract E2 when it was digested at pH 2 may be related to the drastic degradation observed for oleuropein, verbascoside, and other non-abundant phenolic compounds such as flavones [47,48]. However, the digested extract E2 at pH 5 showed a significant (*p* < 0.05) growth reduction of 0.5 log CFU.

## 4. Conclusions

The results obtained in this work demonstrated that in vitro gastric digestion produced changes in the chemical composition of olive leaf extracts (E1 and E2), which also resulted in the modification of their bioactive properties. In extract E1, which contained hydroxytyrosol and its glucoside derivatives as major components, gastric digestion at fasted state (pH 2) produced a significant decrease in the content of these compounds. This behavior also resulted in a decrease in all studied bioactive properties of extract E1 against *H. pylori* (anti-inflammatory, antioxidant, and antibacterial activities). Gastric digestion at fed state (pH 5) had little influence on the composition of extract E1, showing an anti-inflammatory and antioxidant activity similar to non-digested extract E1, but impairing antibacterial activity. Regarding extract E2, which contained oleuropein as a major component, gastric digestion at fasted state (pH 2) showed a similar degradation of identified compounds than that observed in extract E1. This fact resulted in an increase of anti-inflammatory and antioxidant activity, but led to a decrease in antibacterial activity. Meanwhile, digestion of extract E2 at fed state (pH 5) showed a similar behavior to non-digested extract E2. Although there are differences in the composition of both studied extracts, the obtained results have shown that hydroxytyrosol and oleuropein are key components when considering the effectiveness of olive leaf extracts against *H. pylori* with increased resistance to gastric digestion. This information should be considered in the development of extracts with bioactive properties against this pathogen. Finally, these results suggest the most interesting intake patterns to take full advantage of the effectiveness of the assayed olive leaf extracts. Such intake pattern would be different for extracts E1 and E2, depending on the specific bioactivity. Nevertheless, further studies would be necessary to confirm these findings, especially in the case of the complex conditions of fed state, since after ingestion, gastric pH increases up to a range of 5–7, depending on characteristics of the co-existing meals, as well as the buffering capacity or the solid/liquid state.

## Figures and Tables

**Table 1:** Content of identified phenolic and secoiridoid compounds and their derivatives present in the non-digested (E1 and E2) and gastric digested extracts (DE1 and DE2) at fasted (pH 2) and fed state (pH 5) conditions. Results are expressed as mean value ± standard deviation (mg/100 g of dry matter).

Compounds	E1	DE1-pH2	DE1-pH5	E2	DE2-pH2	DE2-pH5
**Hydroxybenzoic acids and glycoside derivatives**						
3,4-DHBA (protocatechuic acid)	11.4 ± 1.0 ^B^	7.3 ± 0.1 ^A^	13.3 ± 0.1 ^B^	6.2 ± 0.2 ^b^	4.8 ± 0.2 ^a^	5.5 ± 0.1 ^b^
3,4-DHBA glucoside	9.2 ± 0.2 ^B^	7.9 ± 0.1 ^A^	9.4 ± 0.3 ^B^	ND	ND	ND
3,4,5-THBA (gallic acid)	ND	86.1 ± 0.9 ^B^	37.2 ± 1.9 ^A^	ND	ND	ND
3,4,5-THBA glucoside 1 (galloyl glucoside 1)	24.0 ± 0.1 ^B^	ND	23.4 ± 0.1 ^A^	ND	ND	ND
3,4,5-THBA glucoside 2 (galloyl glucoside 2)	23.2 ± 0.1 ^B^	ND	22.2 ± 0.1 ^A^	ND	ND	ND
Σ Hydroxybenzoic acids and glycoside derivatives	67.8	101	105	6.2	4.8	5.5
**Hydroxycinnamic acids and** **derivatives**						
*trans*-3,4-DHCA (*trans*-caffeic acid)	140 ± 4 ^B^	117 ± 8 ^A^	140 ± 1 ^B^	7.4 ± 0.2 ^b^	6.7 ± 0.1 ^a^	6.8 ± 0.1 ^a,b^
*trans*-4-HCA (*trans*-4-coumaric acid)	177 ± 1 ^B^	132 ± 11 ^A^	180 ± 1 ^B^	3.2 ± 0.4 ^b^	1.9 ± 0.1 ^a^	2.0 ± 0.1 ^a^
*trans*-3-M,4-HCA (*trans*-ferulic acid)	113 ± 3 ^B^	88.9 ± 7.9 ^A^	114 ± 1 ^B^	5.4 ± 0.3 ^b^	4.6 ± 0.2 ^a^	4.8 ± 0.2 ^a,b^
*trans*-4,5-DCQA (*trans*-4,5-dicaffeoylquinic acid)	ND	ND	ND	28.0 ± 1.3 ^b^	21.4 ± 0.4 ^a^	28.1 ± 1.1 ^b^
Σ Hydroxycinnamic acids and derivatives	430	338	434	44.0	34.6	41.7
**Phenylethanols and glycoside** **derivatives**						
3,4-DPHG (3,4-dihydroxy-phenylglycol)	38.6 ± 0.2 ^B^	31.8 ± 1.3 ^A^	38.4 ± 0.5 ^B^	20.8 ± 0.3 ^a^	33.9 ± 1.6 ^b^	19.7 ± 0.1 ^a^
3,4-DHPE (hydroxytyrosol) + 3,4-DHPE glucoside 1	13,516 ± 81 ^B^	11,566 ± 21 ^A^	13,496 ± 63 ^B^	196 ± 8 ^a^	2631 ± 66 ^b^	176 ± 1 ^a^
3,4-DHPE glucoside 2 + 3	1040 ± 12 ^B^	837 ± 1 ^A^	1025 ± 4 ^B^	159 ± 7 ^a^	176 ± 1 ^b^	145 ± 1 ^a^
4-HPE (tyrosol)	642 ± 1 ^B^	511 ± 1 ^A^	639 ± 1 ^B^	11.1 ± 0.5 ^a^	30.8 ± 1.4 ^b^	9.6 ± 0.1 ^a^
Σ Phenylethanols and glycoside derivatives	15,237	12,946	15,198	387	2872	350
**Secoiridoids and glycoside** **derivatives**						
EA (elenolic acid)	97.6 ± 3.8 ^C^	79.6 ± 3.7 ^B^	34.5 ± 1.8 ^A^	ND	ND	ND
EA 2-glucoside (oleoside 11-methyl ester)	1407 ± 1 ^B^	1100 ± 8 ^A^	1413 ± 7 ^B^	177 ± 3 ^c^	50 ± 1 ^a^	103 ± 1 ^b^
EMA 2-glucoside (secoxyloganin)	1021 ± 21 ^B^	744 ± 3 ^A^	990 ± 7 ^B^	ND	ND	ND
Σ Secoiridoids and glycoside derivatives	2526	1924	2438	177	50	103
**Secoiridoid phenylethanols**						
3,4-DHPE-EA-glucoside (oleuropein)	346 ± 1 ^B^	228 ± 4 ^A^	342 ± 6 ^B^	21,419 ± 1909 ^b^	7353 ± 24 ^a^	18,431 ± 274 ^b^
4-HPE-EA-glucoside (ligustroside)	147 ± 2 ^B^	87.0 ± 8.0 ^A^	126 ± 7 ^B^	344 ± 4 ^c^	157 ± 6 ^a^	296 ± 7 ^b^
Σ Secoiridoid phenylethanols	493	315	468	21,763	7510	18,727
**Cinnamoyl phenyethanol** **glycoside derivatives**						
3,4-DHPE caffeoyl glucoside (verbascoside)	308 ± 6 ^B^	258 ± 8 ^A^	262 ± 5 ^A^	7255 ± 315 ^c^	5196 ± 202 ^a^	6310 ± 44 ^b^
Σ Cinnamoyl phenylethanol glycoside derivatives	308	258	262	7255	5196	6310
**Flavones**						
Apigenin 6,8-di-C-glucoside	316 ± 9 ^B^	252 ± 21 ^A^	321 ± 2 ^B^	34.3 ± 0.1 ^a^	33.1 ± 0.5 ^a^	33.4 ± 0.2 ^a^
Apigenin 7-*O*-rutinoside (isorhoifolin)	129 ± 1 ^B^	84.7 ± 7.8 ^A^	125 ± 5 ^B^	124 ± 4 ^b^	77.0 ± 1.0 ^a^	116 ± 7 ^b^
Apigenin 7-*O*-glucuronide	70.1 ± 0.5 ^B^	31.0 ± 3.9 ^A^	62.5 ± 11.9 ^B^	115 ± 3 ^c^	30.0 ± 0.3 ^a^	47.4 ± 1.9 ^b^
Luteolin 3′,7-di-*O*-glucoside	39.4 ± 0.3 ^B^	34.5 ± 0.8 ^A^	40.0 ± 0.7 ^B^	31.2 ± 0.4 ^b^	28.2 ± 0.7 ^a^	30.2 ± 0.5 ^a,b^
Luteolin 7-*O*-glucoside	702 ± 2 ^B^	385 ± 6 ^A^	695 ± 7 ^B^	517 ± 11 ^b^	274 ± 4 ^a^	500 ± 4 ^b^
Luteolin 4′-methyl ether 7-*O*-glucoside (diosmin)	84.2 ± 0.1 ^B^	68.4 ± 0.5 ^A^	83.6 ± 0.8 ^B^	85.9 ± 1.5 ^b^	51.7 ± 5.8 ^a^	56.7 ± 4.0 ^a^
Luteolin	ND	ND	ND	17.0 ± 0.5 ^b^	9.55 ± 0.31 ^a^	13.9 ± 2.2 ^a,b^
Σ Flavones	1341	857	1327	924	504	798
**Flavonols**						
Quercetin 3-*O*-glucoside (isoquercitrin)	ND	ND	ND	15.6 ± 0.3 ^b^	6.9 ± 0.3 ^a^	12.8 ± 2.4 ^b^
Quercetin 3-*O*-rhamnoside (quercitrin)	ND	ND	ND	7.4 ± 0.7 ^b^	3.3 ± 0.2 ^a^	6.6 ± 0.9 ^b^
Quercetin	ND	ND	ND	24.2 ± 0.1 ^a^	25.8 ± 0.1 ^b^	24.4 ± 0.1 ^a^
Σ Flavonols	ND	ND	ND	47.2	36.0	43.8
**Flavanones**						
Eriodictyol 7-*O*-rutinoside	ND	ND	ND	14.9 ± 0.1 ^b^	8.3 ± 0.61 ^a^	13.8 ± 0.1 ^b^
Eriodictyol 7-*O*-glucoside	ND	ND	ND	24.5 ± 0.3 ^a^	ND	25.9 ± 0.6 ^a^
Σ Flavanones	ND	ND	ND	39.4	8.3	39.7
Σ Phenolic and secoiridoid compounds	20,403	16,739	20,233	30,643	16,216	26,419

ND: not detected; 3-M,4-HCA: 3-methoxy-4-hydroxycinnamic acid; DCQA: dicaffeoylquinic acid; DHBA: dihydroxybenzoic acid; DHCA: dihydroxycinnamic acid; DHPE: dihydroxyphenylethanol; EMA 2-glucoside: EA monoaldehyde isomer 2-glucoside; HCA: hydroxycinnamic acid; HPE: hydroxyphenylethanol; THBA: trihidroxybenzoic acid. ^A–C^ Values in the same row marked with different uppercase letters indicate significant differences between non-digested and gastric digested E1 extracts by ANOVA *post hoc* Tukey test (*p* ≤ 0.05). ^a–c^ Values in the same row marked with different lowercase letters indicate significant differences between non-digested and gastric digested E2 extracts by ANOVA *post hoc* Tukey test (*p* ≤ 0.05).

**Table 2:** Antibacterial activity of olive leaf extracts (E1 and E2) and their digested samples at pH 2 and pH 5 against *H. pylori*. Results represent the mean ± standard deviation of colony forming units (CFU)/mL (n = 3).

Extracts	CFU/mL	Log CFU Reduction
Control growth	6.33 ± 0.25 × 10^8^	-
E1	1.43 ± 0.25 × 10^3 a^*	1.6
DE1-pH2	1.79 ± 0.28 × 10^8 b^*	0.5
DE1-pH5	1.81 ± 0.15 × 10^8 b^*	0.5
E2	1.44 ± 0.11 × 10^8 a^*	0.6
DE2-pH2	5.13 ± 1.31 × 10^8 b^	0.1
DE2-pH5	2.13 ± 0.38 × 10^8 a^*	0.5

Detection limit was 1.5 log CFU/mL (30 cfu per plate). * Values marked with asterisk indicates significant differences compared to the control growth by *t*-test (*p* < 0.05). ^a,b^ Different letters indicate significant differences within a same extract by ANOVA post hoc HSD Tukey test (*p* < 0.05).

## Data Availability

The data presented in this study are available in this manuscript.

## Supplementary Materials

The following are available online at https://www.mdpi.com/article/10.3390/foods11131832/s1, Figure S1: HPLC chromatogram of olive leave extract E1 and its gastric digests. Figure S2: HPLC chromatogram of olive leave extract E2 and its gastric digests
