# Supplementary material for: Influence of In Vitro Gastric Digestion of Olive Leaf Extracts on Their Bioactive Properties against H. pylori
Source: Foods. 2022 Jun 22;11(13):1832. doi: 10.3390/foods11131832 (PMC9265983; doi:10.3390/foods11131832)
Supplement: Supplementary file 1 [file foods-11-01832-s001.zip › foods-1747521-supplementary.pdf]

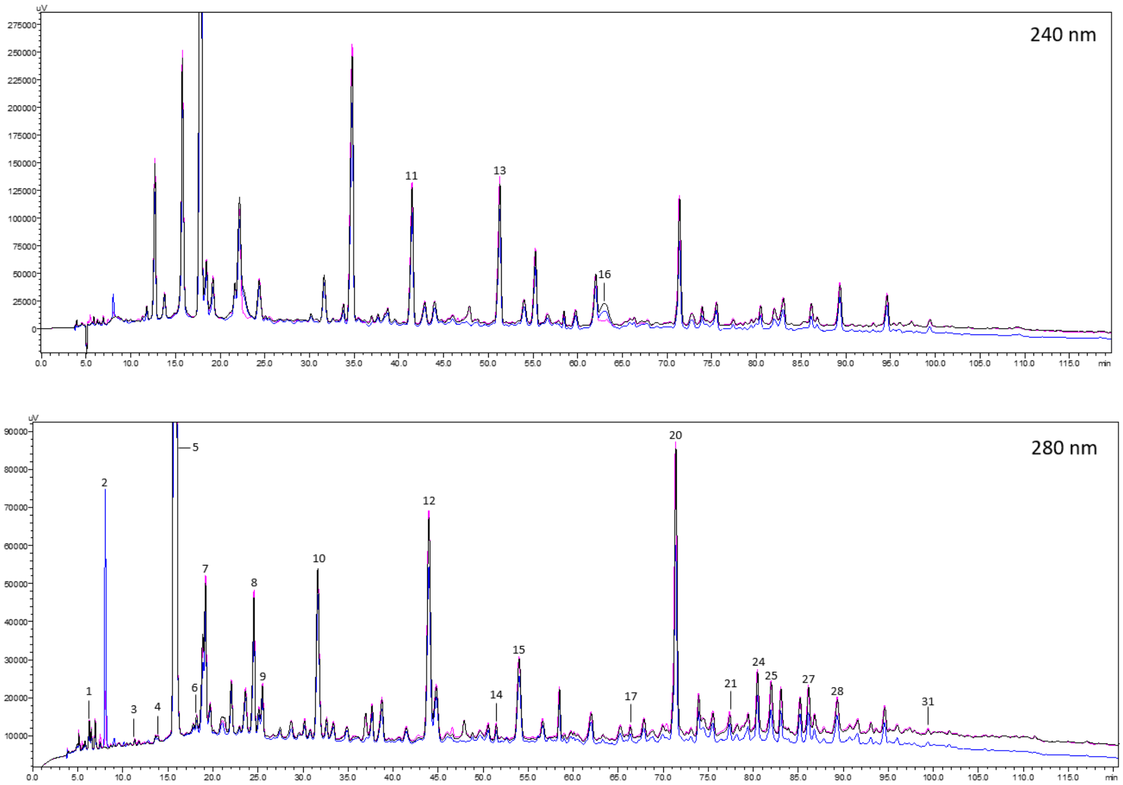

Supplementary figure S1. HPLC chromatogram of olive leave extract E1 (black trace) and its gastric digests, DE1-pH2 (blue trace) and DE1-pH5 (red trace), at 240 nm (upper chromatogram) and 280 nm (lower chromatogram), (1) 3,4-DPHG, (2) 3,4,5-THBA, (3) 3,4-DHBA glucoside, (4) 3,4-DHBA, (5) 3,4-DHPE + 3,4-DHPE glucoside 1, (6) 3,4,5-THBA glucoside 1, (7) 3,4-DHPE glucoside 2+3, (8) 4-HPE, (9) 3,4,5-THBA glucoside 2, (10) *trans*-3,4-DHCA, (11) EA 2-glucoside, (12) *trans*-4-HCA, (13) EMA 2-glucoside, (14) Apigenin 6,8-di-C-glucoside, (15) *trans*-3-M,4-HCA, (16) EA, (17) Luteolin 3',7-di-O-glucoside, (20) Luteolin 7-O-glucoside, (21) 3,4-DHPE caffeoyl glucoside, (24) Apigenin 7-O-rutinoside, (25) Apigenin 7-O-glucuronide, (27) Luteolin 4'-methyl ether 7-O-glucoside, (28) 3,4-DHPE-EA glucoside, (31) 4-HPE-EA-glucoside.

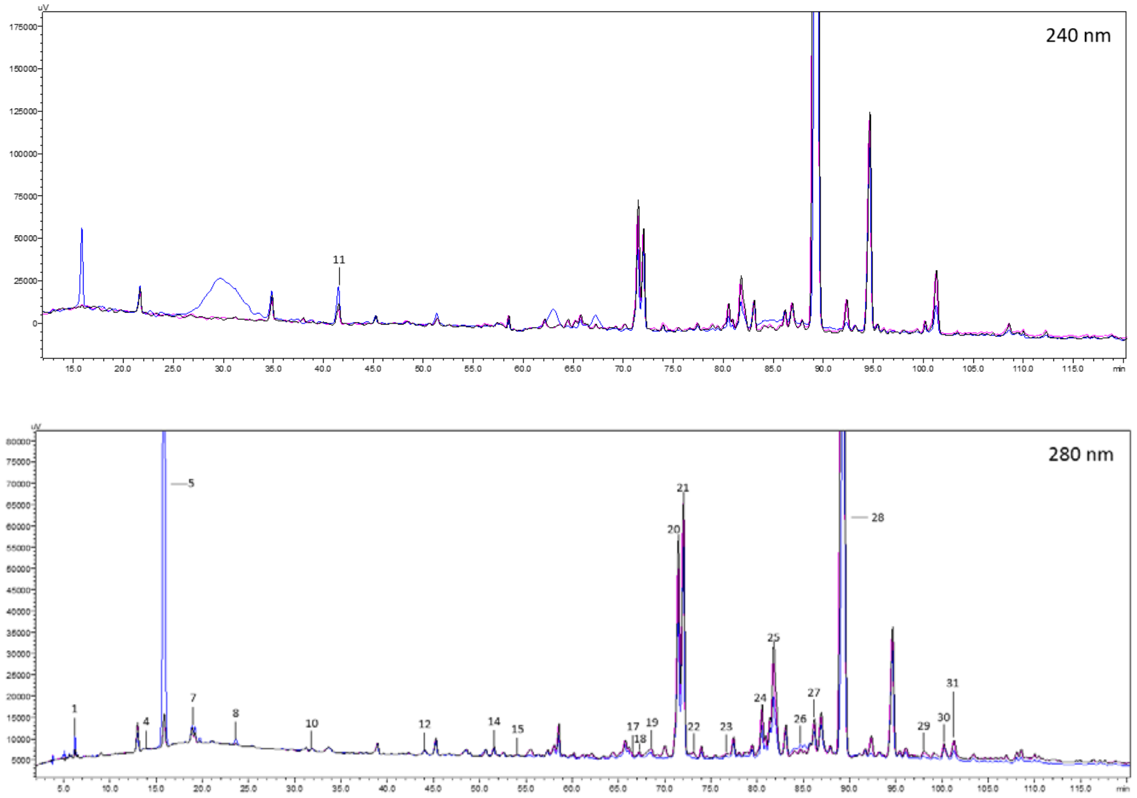

Supplementary figure S2. HPLC chromatogram of olive leave extract E2 (black trace) and its gastric digests, DE2-pH2 (blue trace) and DE2-pH5 (red trace), at 240 nm (upper chromatogram) and 280 nm (lower chromatogram), (1) 3,4-DPHG, (4) 3,4-DHBA, (5) 3,4-DHPE + 3,4-DHPE glucoside 1, (7) 3,4-DHPE glucoside 2+3, (8) 4-HPE, (10) *trans*-3,4-DHCA, (12) *trans*-4-HCA, (14) Apigenin 6,8-di-C-glucoside, (15) *trans*-3-M,4-HCA, (17) Luteolin 3',7-di-O-glucoside, (18) Quercetin 3-O-glucoside, (19) Eriodictyol 7-O-rutinoside, (20) Luteolin 7-O-glucoside, (21) 3,4-DHPE caffeoyl glucoside, (22) Eriodictyol 7-O-glucoside, (23) Quercetin 3-O-rhamnoside, (24) Apigenin 7-O-rutinoside, (25) Apigenin 7-O-glucuronide, (26) *trans*-4,5-DCQA, (27) Luteolin 4′-methyl ether 7-O-glucoside, (28) 3,4-DHPE-EA glucoside, (29) Luteolin, (30) Quercetin, (31) 4-HPE-EA-glucoside.
